# Does comorbidity index predict OPAT readmission?

**DOI:** 10.1093/jacamr/dlad125

**Published:** 2023-11-23

**Authors:** Ryan D Stubbs, Robert J Shorten, Valerio Benedetto, Alison Muir

**Affiliations:** Faculty of Biology, Medicine and Health, University of Manchester, Oxford Road, Manchester M13 9PL, UK; Department of Microbiology, Lancashire Teaching Hospitals NHS Foundation Trust, Sharoe Green Lane, Preston PR2 9HT, UK; Applied Health Research hub, University of Central Lancashire, Victoria Street, Preston PR1 2HE, UK; Department of Microbiology, Lancashire Teaching Hospitals NHS Foundation Trust, Sharoe Green Lane, Preston PR2 9HT, UK

## Abstract

**Objectives:**

To determine if the Charlson comorbidity index (CCI) is an accurate predictor of unplanned readmissions for patients using outpatient parenteral antimicrobial therapy (OPAT) services.

**Methods:**

Retrospective analysis of patients >16 years of age who had received OPAT at Lancashire Teaching Hospitals between 2019 and 2021. The number of unplanned hospitalizations was measured and categorized as OPAT related or non-OPAT related. The CCI for each patient group was calculated using an online tool, and logistic regression was used to assess the association between risk factors and risk of being readmitted.

**Results:**

The cohort consisted of 741 patients. Unplanned readmission was seen in 112 patients (15.1%). The mean CCI score for patients with OPAT-related readmissions was 4.22, 0.92 higher than the mean for patients who were not readmitted (3.30). The mean CCI score for patients with non-OPAT-related readmissions was higher still at 4.89. The logistic regression showed that increased CCI, age, male gender and home location compared with clinic were associated with increased odds of readmission, although these effects did not meet statistical significance.

**Conclusions:**

These results suggest that a higher CCI score is associated with a non-statistically significant increased risk of unplanned hospitalization. We concluded that the CCI may therefore be used in future decision-making regarding the acceptance of patients to OPAT and requires further investigation.

## Introduction

Outpatient parenteral antimicrobial therapy (OPAT)—providing IV antimicrobial therapy to patients in a clinic or home-based setting—is now a standard aspect of UK healthcare.^1^ However, like all healthcare interventions, OPAT is not risk free; one Canadian study showed that approximately 26% of patients experienced a readmission during their treatment.^2^ Complication and readmission rates in a UK study were 6.4%.^3^ More recently, Keller *et al*.^4^ showed that patients receiving home OPAT for longer than 28 days, and patients who received vancomycin, had increased risks of developing adverse outcomes. Gilchrist *et al*.^5^ summarized findings from the UK National OPAT Registry. This revealed considerable variation between OPAT services in the proportions of episodes classed as infection failure and OPAT success. Overall, 90.8% of patients had OPAT outcomes classed as success/partial success. However, there was an increased risk of OPAT failure for patients with urinary/genitourinary tract infections and bronchiectasis. There is limited published literature on the predictors of readmission for OPAT patients. Two US studies have attempted to evaluate risk factors for unplanned hospitalizations.^6,7^ Schmidt *et al*.^6^ showed that the location of OPAT administration was associated with unplanned hospitalization. They also demonstrated that there was a significant difference between the Charlson comorbidity index (CCI) scores for patients who were readmitted and those who were not. However, Allison *et al*.^7^ found that CCI did not have a statistically significant effect on 30 day readmissions for OPAT.

The CCI was developed in 1987 as a weighted index to predict 10 year survival in patients with multiple comorbidities.^8^ Each comorbidity increases the score, and the higher the score, the lower the estimated chance of survival in the next 10 years.^8^

This service improvement project was undertaken to determine if there was an association between CCI and risk of readmission for our UK teaching hospital OPAT patients, as increased readmission rates had been noted in our home OPAT cohort, who are generally older/frailer and with more comorbidities than the clinic OPAT patients. We also explored whether additional factors including indication for OPAT, antibiotic used, treatment duration and line complications were linked to increased risk of readmission. Using this information, we hope to be able to improve patient selection and risk assessment for OPAT.

## Methods

This study took place at Lancashire Teaching Hospitals NHS Foundation Trust (LTH), a large secondary care trust in the Northwest of England, which provides tertiary services including vascular surgery, neurosurgery and oncology. It offers both home and clinic-based OPAT. The clinic OPAT service was piloted in 2012 using an ambulatory model with patients attending a day unit on the hospital site for daily treatment and OPAT team review. The home OPAT service was established in 2017 and is run in conjunction with the local district nursing teams who visit to provide IV antibiotic administration, line care and blood sampling with oversight from the OPAT team/multidisciplinary team (MDT).

All referrals to the OPAT service must be approved by a consultant microbiologist/infection specialist. Patients are then holistically reviewed by the OPAT team to determine suitability for the service and the most appropriate form of OPAT. The key factors that lead to a patient being treated via home OPAT include frailty, poor mobility and difficulty reaching the hospital site (physical, geographical and social).

Retrospective analysis of data routinely collected for clinical care and outcome monitoring of patients receiving OPAT at LTH between 2019 and 2021 was undertaken. Demographic data, indication for antimicrobial therapy, antimicrobial agent(s) prescribed, duration of therapy (from initiation of OPAT to either completion of IV therapy or readmission) and OPAT location were recorded. Adverse events, reason for readmission, OPAT and patient outcomes were also recorded in accordance with the BSAC Good Practice Recommendations.^2,9^ OPAT-related readmissions were defined as those associated with the infection or antimicrobial therapy, such as treatment failure, drug reactions or line complications. Readmissions for unrelated reasons were classed as non-OPAT related.

The CCI was calculated using MDCalc from conditions that the patient had been diagnosed with at the time of acceptance to the OPAT service.^10^

### Statistical analysis

ANOVA was used to compare the difference between all three categories (OPAT-related readmissions, non-OPAT-related readmissions, and no readmission) for continuous variables such as mean CCI. A chi-squared test was used to compare binary/nominal variables such as antibiotic used and infusion location. The Kruskal–Wallis test was used for ordinal variables such as age and treatment duration. The significance level was set at 0.05, and each was performed as a two-tailed test.

We also developed a logistic regression model to estimate the strength and direction of the associations between a set of demographic and clinical variables and the odds of being readmitted. In particular, the model included OPAT location, gender, age, OPAT indication, antibiotic to be given, OPAT bed-days and PICC line complication as independent variables, with the statistical significance set at the 5% level for *P* values.

## Ethics

This study was determined to be a local service evaluation project and ethics approval was not required.

## Results

Seven hundred and forty-one patients received OPAT during 2019–21 and none were excluded from analysis. Most patients were over 60 years of age (446; 60.2%) and had a treatment duration of 42 days or less (695; 93.8%). Median treatment duration for the whole cohort was 13 days (IQR 6–21), for home OPAT was 14 days (IQR 5–24) and for clinic OPAT was 10 days (IQR 7–19). The most common single indication for OPAT was intra-abdominal abscess (202; 27.3%). More patients received clinic OPAT than home OPAT (497; 67.1% versus 244; 32.9%). The most frequently prescribed antibiotic was ceftriaxone (417/741; 56.3%). The most common CCI score was 2–3 (Table 1).

**Table 1.** Characteristics of patients prescribed OPAT between 2019 and 2021 in the study cohort

Variable	Overall cohort (*n* = 741)	No unplanned readmission (*n* = 629)	OPAT-related readmission (*n* = 49)	Non-OPAT-related readmission (*n* = 63)	*P* value
Gender, *n* (%)					0.697
* *Male	385 (52.0)	323 (51.4)	28 (57.1)	34 (54.0)	
* *Female	356 (48.0)	306 (48.6)	21 (42.9)	29 (46.0)	
Age (years), *n* (%)					<0.001
* *17–29	32 (4.3)	31 (4.9)	1 (2.0)	0 (0.0)	
* *30–39	54 (7.3)	49 (7.8)	3 (6.1)	2 (3.2)	
* *40–49	74 (10.0)	68 (10.8)	3 (6.1)	3 (4.8)	
* *50–59	135 (18.2)	125 (19.9)	7 (14.3)	3 (4.8)	
* *60–69	171 (23.1)	148 (23.5)	6 (12.2)	17 (27.0)	
* *70–79	189 (25.5)	140 (22.3)	22 (44.9)	27 (42.9)	
* *80–89	78 (10.5)	61 (9.7)	7 (14.3)	10 (15.9)	
* *>90	8 (1.1)	7 (1.1)	0 (0.0)	1 (1.6)	
Treatment duration (days), *n* (%)					0.255
* *<14	379 (51.1)	311 (49.4)	31 (63.3)	37 (58.7)	
* *14–42	316 (42.6)	286 (45.5)	10 (20.4)	20 (31.7)	
* *>42	46 (6.2)	32 (5.1)	8 (16.3)	6 (9.5)	
CCI mean					<0.001
* *CCI mean at discharge	3.48	3.28	4.22	4.89	
CCI, *n* (%)					<0.001
* *0–1	186 (25.1)	174 (27.7)	8 (16.3)	4 (6.3)	
* *2–3	207 (27.9)	179 (28.5)	16 (32.7)	12 (19.0)	
* *4–5	182 (24.6)	153 (24.3)	8 (16.3)	21 (33.3)	
* *6–7	98 (13.2)	73 (11.6)	9 (18.4)	16 (25.4)	
* *>7	68 (9.2)	50 (7.9)	8 (16.3)	10 (15.9)	
Indication for OPAT, *n* (%)					0.016
* *Intra-abdominal abscess	202 (27.3)	161 (25.6)	22 (44.9)	19 (30.2)	
* *Bronchiectasis	128 (17.3)	112 (17.8)	3 (6.1)	13 (20.6)	
* *Cellulitis	127 (17.1)	120 (19.1)	7 (14.3)	0 (0.0)	
* *Discitis	35 (4.7)	27 (4.3)	2 (4.1)	6 (9.5)	
* *Empyema	37 (5.0)	28 (4.5)	1 (2.0)	8 (12.7)	
* *Endocarditis	21 (2.8)	20 (3.2)	0 (0.0)	1 (1.6)	
* *Prosthetic joint infection	62 (8.4)	53 (8.4)	2 (4.1)	7 (11.1)	
* *Osteomyelitis	32 (4.3)	27 (4.3)	3 (6.1)	2 (3.2)	
* *Other	84 (11.3)	70 (11.1)	7 (14.3)	7 (11.1)	
* *Native joint septic arthritis	13 (1.8)	11 (1.7)	2 (4.1)	0 (0.0)	
OPAT infusion location, *n* (%)					<0.001
* *Home OPAT	244 (32.9)	186 (29.6)	20 (40.8)	38 (60.3)	
* *Clinic OPAT	497 (67.1)	443 (70.4)	29 (59.2)	25 (39.7)	
Antibiotic given, *n* (%)					<0.001
* *Aztreonam	19 (2.6)	18 (2.9)	0 (0.0)	1 (1.6)	
* *Ceftriaxone	417 (56.3)	372 (59.1)	25 (51.0)	20 (31.7)	
* *Ertapenem	86 (11.6)	64 (10.2)	7 (14.3)	15 (23.8)	
* *Other	31 (4.2)	26 (4.1)	2 (4.1)	3 (4.8)	
* *Piperacillin/tazobactam	116 (15.7)	96 (15.3)	4 (8.2)	16 (25)	
* *Teicoplanin	59 (8.0)	46 (7.3)	6 (12.2)	7 (11.1)	
* *Tigecycline	13 (1.8)	7 (1.1)	5 (10.2)	1 (1.6)	
Issues with PICC Line requiring removal, *n* (%)	31 (4.2)	23 (3.7)	4 (8.2)	4 (6.3)	0.211

The values are displayed as a percentage of the total cohort for each row. *P* values calculated using the ANOVA test for continuous variables, using the chi-squared test for binary/nominal variables, and using the Kruskal–Wallis test for ordinal variables.

Unplanned readmissions were uncommon (112/741; 15.1%). Of the 112 readmissions, 63 (8.5% of the total cohort) were non-OPAT related, 49 (6.6% of the total cohort) were OPAT related. Readmission was more frequent in patients aged over 69 years. The lowest rates of readmission were seen in patients aged 17–39 years (Table 1). The median time to readmission was 11 days (IQR 4–20).

The CCI was lower on average for those who were not readmitted (3.28). Mean CCI score for patients with OPAT-related readmissions was higher at 4.22, and patients experiencing non-OPAT-related readmissions had the highest mean CCI at 4.89 (*P*  ≤  0.001) (Table 1).

The spread of CCI scores for the cohort was also compared with the frequency of admissions. Patients with CCI scores of 0–1 were less likely to be readmitted, whilst those with a score of 2–3 were less likely to be readmitted for non-OPAT-related causes. Patients had a higher chance of being readmitted if their CCI was 6 or above (Table 1).

Patients with intra-abdominal abscess were most likely to be readmitted, and also made up the largest proportion of OPAT-related readmissions (22/49; 44.9%). Those with endocarditis, empyema, discitis, native joint septic arthritis or prosthetic joint infections had the lowest rates of OPAT-related readmissions. For non-OPAT-related readmissions, patients with intra-abdominal abscess and bronchiectasis were most likely to be readmitted, whilst those with cellulitis and native joint septic arthritis were least likely to be affected.

Receiving OPAT at home was significantly associated with a higher rate of readmission. There was a total of 58 readmissions from home OPAT (23.8% of home OPAT patients), whereas for clinic OPAT there were 54 readmissions (10.9% of clinic OPAT patients). Even though home OPAT patients comprised only 32.9% of the total cases, they contributed to 51.8% of the total readmissions, giving a risk ratio of 1.57. Further analysis of the relationship between home and clinic OPAT was performed and showed that the mean CCI was significantly different between the two groups: clinic OPAT 2.9, home OPAT 4.63, cohort mean 3.48. Comparison of age profiles demonstrated that home OPAT patients were generally more elderly, with 83.2% aged 60 years or older, compared with 48.9% of clinic OPAT patients. It was also found that a larger proportion of patients with intra-abdominal abscess received treatment in clinic (140/202; 69.3%).

Ceftriaxone was the most frequently used antibiotic in readmitted patients (45/112) and for both OPAT- and non-OPAT-related reasons (25/49; 51.0% and 20/63; 31.7%, respectively). However, when considering the chance of readmission by antimicrobial used, patients receiving ceftriaxone had the lowest risk (45/417; 10.8%). Patients receiving ertapenem and piperacillin/tazobactam had a higher chance of being readmitted for non-OPAT-related reasons. Patients administered teicoplanin and tigecycline had higher rates of OPAT-related readmission.

Ceftriaxone was used more frequently for clinic OPAT patients, making up 61.4% of antibiotic usage for clinic OPAT, in comparison with 43.4% in home OPAT.

The results from the logistic regression are presented in Table 2, based on 741 observations. This only showed a statistically significant effect for patients with cellulitis who had a lower risk of readmission with respect to the reference category (i.e. being affected by intraabdominal abscess) in contrast with those with other infections. Home location compared with clinic, male gender, each additional year of age and higher CCI were associated with increased odds of readmission. For infection diagnosis and antimicrobial, patients with empyema and those receiving tigecycline had the highest odds of readmission. Line complication also increased the risk of readmission but none of these effects met statistical significance. Treatment duration did not affect the risk of readmission.

**Table 2.** Logistic regression on risk of readmission

*n* = 741 Pseudo R^2^ = 0.11	OR (standard error)	*P* value	95% CI
OPAT location			
Clinic (reference category)	—	—	—
Home	1.23 (0.31)	0.411	0.751–2.010
Gender			
Female (reference category)	—	—	—
Male	1.13 (0.26)	0.571	0.733–1.756
Age (years)	1.02 (0.01)	0.068	0.998–1.044
OPAT indication			
Intrabdominal abscess (reference category)	—	—	—
Bronchiectasis	0.38 (0.19)	0.052	0.139–1.010
**Cellulitis**	**0.31** (**0.15)**	**0**.**014**	**0.125–0.789**
Discitis	1.05 (0.52)	0.929	0.395–2.763
Empyema	1.27 (0.59)	0.615	0.505–3.177
Endocarditis	0.15 (0.17)	0.084	0.018–1.289
Prosthetic join infection	0.46 (0.21)	0.085	0.195–1.110
Osteomyelitis	0.64 (0.36)	0.431	0.211–1.945
Other	0.61 (0.23)	0.192	0.293–1.280
Native joint septic arthritis	0.77 (0.64)	0.752	0.151–3.925
CCI	1.11 (0.06)	0.081	0.988–1.240
Antibiotic to be given			
Aztreonam (reference category)	—	—	—
Ceftriaxone	2.32 (2.65)	0.461	0.247–21.825
Ertapenem	4.82 (5.58)	0.173	0.501–46.495
Other	4.97 (6.15)	0.196	0.438–56.243
Piperacillin/tazobactam	4.25 (4.55)	0.177	0.520–34.645
Teicoplanin	5.34 (6.32)	0.157	0.525–54.350
Tigecycline	11.15 (14.34)	0.061	0.897–138.568
OPAT bed-days	1.00 (0.01)	0.810	0.985–1.011
PICC line complication			
No (reference category)	—	—	—
Yes	1.21 (0.62)	0.703	0.448–3.294

Bold type indicates statistical significance.

## Discussion

This study has demonstrated factors associated with an increased risk of unplanned readmission in our setting. It has provided insight into the use of the CCI when assessing the risks of referring a patient to OPAT. To our knowledge, this is the first UK study to investigate the relationship between CCI and readmission rates.

Multivariate analysis showed that increased CCI, age, male gender and home location compared with clinic were associated with increased odds of readmission, although these effects did not meet statistical significance.

The finding of an association between CCI and risk of readmission has previously been demonstrated by Luu *et al*.^11^ and Schmidt *et al*.^6^ Schmidt *et al.*^6^ found lower rates of readmission in patients treated at home compared with skilled nursing facilities, but this study was conducted in the USA, where healthcare models differ. This contrasts with the work of Allison *et al*.,^7^ who reported that CCI did not affect 30 day OPAT readmissions.

Multivariate analysis showed that patients with cellulitis had statistically significant decreased chance of readmission compared with other infections, and this warrants further investigation.

Calculating CCI is straightforward and could be used as part of the patient assessment and selection process for OPAT. CCI may help to guide and inform discussions involving the parent medical team, OPAT team and patient regarding the risks/benefits/suitability of alternative infection management options and to manage expectations regarding their relative outcomes. It may also open useful dialogue concerning optimization for comorbid conditions prior to commencement of OPAT. A prospective evaluation of CCI in the OPAT service is now underway.
